# Nanoscale Mechanical and Morphological Characterization of Ebolavirus-like Particles: Implications for Therapeutic Development

**DOI:** 10.3390/ijms26115185

**Published:** 2025-05-28

**Authors:** Hannah Hargrove, Susana A. Torres-Hurtado, Wendy J. Maury, Xiaohui Frank Zhang

**Affiliations:** 1 Department of Biomedical Engineering and Department of Chemical Engineering, University of Massachusetts Amherst, Amherst, MA 01003, USA; satorresh@umass.edu; 2 Department of Microbiology and Immunology, University of Iowa, Iowa City, IA 52242, USA; wendy-maury@uiowa.edu

**Keywords:** Ebolavirus, EBOVΔVP30, EBOVΔVP40 VLP, rVSV-EBOV-GP, virus-like particles (VLPs), atomic force microscopy (AFM) imaging, atomic force spectroscopy, elastic modulus nanoindentation

## Abstract

Zaire Ebolavirus (EBOV) is one type of filovirus that causes the deadly EBOV disease, with an average fatality rate of around 50%. EBOV outbreaks are devastating and unpredictable and may emerge as the next global pandemic. As a BSL-4 pathogen, EBOV is inaccessible to regular biological laboratories. Therefore, EBOV virus-like particles (EBOV-VLPs) and EBOV pseudoviruses (EBOV-PVs) are utilized in the initial development of many potential therapies, for safety reasons and ease of procurement, as opposed to using infectious viruses. To investigate the host cell entry of EBOV and develop viral entry blockers, the EBOV model virions must accurately represent the morphological and mechanical properties of infectious EBOV virions. Due to the nanometer scale and irregular shape of EBOVs, these properties are challenging to characterize. In this research, state-of-the-art nanoscale characterization techniques are employed to examine the mechanical and structural elements of a selection of commonly used EBOV-approximating model virions. This study comprehensively determines the accuracy of EBOV approximation with a variety of model virions and the uniformity of mechanical and structural traits across different model virion types and preparation methods. This provides important implications for developing therapeutic treatments against EBOV using these model virions.

## 1. Introduction

Filoviruses, such as Marburg (MARV) and Ebola (EBOV) viruses, are negative-sense, lipid-enveloped RNA viruses with long, thread shaped virions that can take multiple configurations [1]. The filamentous conformation can be long, short, branched, unbranched, circular, or forming “6” and “U” configurations [2]. The fatality of these viruses can be up to 90% [1,3,4], and there are currently only two FDA-approved antiviral therapies against EBOV [5]. Based on CDC classification, EBOV is considered to be a biosafety level 4 pathogen, and is regarded as a category A bioterrorism weapon [6]. This means that in an outbreak, the EBOV pathogen has an extremely high likelihood for extensive transmission within a population. This was observed during the first Zaire EBOV outbreak, in 1976, where 318 human cases resulted in deaths (88% mortality). In general, the number of infected subjects has decreased as diagnostics for EBOV improved and as quarantine and treatment procedures became more refined. However, the mortality of EBOV is still incredibly high—in the 2014 outbreak in the Democratic Republic of the Congo, 66 people were infected and 49 died (74% mortality) [6]. This is reflective of the fact that to date, EBOV disease management mainly relies on supportive and symptomatic therapy, rather than specialized antiviral treatments [6]. In light of the high mortality of this disease, coupled with the limited availability and specificity of current treatments, it is critical to focus on the development of more effective antiviral therapies and vaccines, in order to reduce this mortality percentage.

The study of infectious EBOV is constrained to BSL4 facilities. Therefore, accurate approximations must be made to study the effects of the virus in a lower-level BSL-rated lab, and characterization of EBOV model virions is a critical target for the development of effective therapies. The EBOV virion can rely on a wide variety of attachment factors to enter the host cell. In enveloped viruses like EBOV, the VP40 matrix is surrounded by a lipid bilayer that contains viral glycoproteins (GPs). In some cells, the presence of phosphatidylserine (PS) on the viral envelope enhances the internalization of the virus through T-cell immunoglobulin and mucin domain (TIM) binding to PS. TIMs are type I transmembrane glycoproteins with one Ig-like V domain. Both TIM-1 and TIM-4 proteins are known to mediate EBOV entry via binding of PS, along with a wide selection of other GP and PS receptors. Thus far, no receptors have appeared to be uniquely critical to EBOV entry. This versatility makes it difficult to develop a therapy that can fully prevent viral entry [2]. After binding to a host cell receptor takes place, EBOV can enter the cell in one of three mechanisms: macropinocytosis, clathrin-mediated endocytosis, and caveolin-mediated endocytosis [2]. Currently, macropinocytosis is believed to be the dominant uptake mechanism [2]. In addition to this, it appears that the dominant internalization method is dependent on the physical conformation of the virus, including their shapes and mechanical properties. One of the significant surface features of EBOV is the GP, which is composed of GP1 and GP2. After uptake, GP1 is proteolytically processed within the endosome and then binds to NPC1 [2]. Additionally, GP is responsible for the pathogenic differences in different strains of EBOV [2], making its presence a critical component of characterizing EBOV model virions.

If the research is being conducted in a lab with a BSL-4 rating, then infectious or unaltered EBOV virus might be used. This has a high degree of risk and requires special training and equipment to handle. Due to the high safety requirements for the lab and the high level of risk to the scientist, infectious EBOV cannot be studied by most labs. Instead, the average researcher uses an EBOV approximating virion to model the behavior of infectious EBOV. In this study, an altered version of EBOV, referred to as EBOVΔVP30, is used as a reference point for the mechanical and structural properties of EBOV. This VP30-null virion is used as a model virus for studying EBOV. The VP30 gene segment is replaced with GFP, rendering the resulting particle capable of replicating only in cells that express VP30. The physiological properties of this virion are likely to be very similar to infectious EBOV.

Recombinant vesicular stomatitis virus (rVSV) is a Rhabdovirus used extensively as a viral platform for studying the viral GPs of high-containment viruses since rVSV readily incorporates a wide range of viral GPs onto virions [7,8,9]. Within the genome, the native GP of VSV (G) is replaced by EBOV GP and the GFP reporter genes in this recombinant virus model system.

As VSV PVs have a distinctly different particle shape and may be deemed to be less appropriate for the study in question, alternative approximating EBOV virus like particles (VLPs) can also be studied. Unlike PVs, VLPs are specially engineered particles designed to mimic viruses. In the case of EBOV VLPs, these lack a viral genome and are composed of EBOVVP40 and EBOV GP. EBOV NP is sometimes added to enhance the stability of the virion [10].

The filamentous nature of EBOV was first observed via transmission electron microscopy in 1976 by Frederick A. Murphy [11]. However, while the surface components of EBOV have been well characterized to date [12], the biomechanical properties of the EBOV virion have not been fully characterized before now. In this study, atomic force microscopy (AFM) is utilized to quantify the mechanical and biochemical properties of rVSV-EBOV-GP and an EBOV VLP in comparison to a sample of EBOVΔVP30. Each of these model virions are meant to represent the physical and biomechanical properties of EBOV. As such, the properties of rVSV-EBOV-GP and the EBOV VLP are compared to EBOVΔVP30 and the physical properties of EBOV taken from published reports.

## 2. Results

### 2.1. Physical Conformation of the Samples

**Figure 1:**
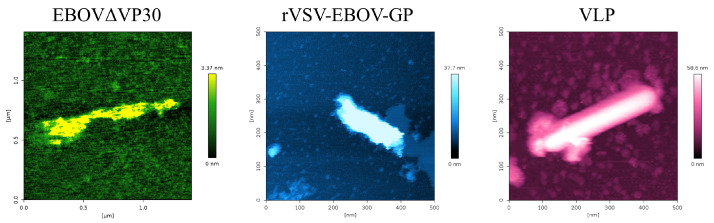
PeakForce Tapping^®^ mode AFM images of each sample imaged under 1xPBS. High-resolution images acquired with PEAKFORCE-HIRS-F-A tip, which has a nominal tip radius of 1 nm. For a 3D representation of each particle, see Supplemental Videos S1–S3.

When attempting to study EBOV in a BSL-1 lab, it is impossible to use infectious EBOV. The closest viral preparation available to us is EBOVΔVP30, followed by rVSV-EBOV-GP, then by synthesized EBOV approximations like EBOV VLP. A sample image of each of these particles can be seen in Figure 1. Each of these particles was adhered to mica via a poly-L-lysine coating, and then imaged via PeakForce Tapping^®^ Imaging. Based on tapping mode scans, the EBOVΔVP30 particles appeared as a wide variety of shapes, ranging from spherical virions to long, filamentous virions that exhibited a high degree of conformational bending. Conversely, the rVSV-EBOV-GP and VLP samples demonstrated a more uniform shape, with shorter, more capsule-shaped configurations. This is expected of the rVSV-EBOV-GP, as rVSV is naturally bullet-shaped [13]. Physically, the Sf9 EBOV VLP envelope is more closely aligned with the shape of the rVSV-EBOV-GP sample than that of the EBOVΔVP30 sample. Additionally, the height profile of each virion was observed to have a squat, domed shape while adhered to the mica substrate, with the profile of each being slightly wider and shallower than expected—this is likely due to the virion deforming slightly in order to contact more of the surface. This could also be due in part to the force-based imaging technique, which is likely depressing each sample slightly. This force-based imaging method may also explain slight variations in virion height.

When imaging EBOVΔVP30 for topographical analysis, many filamentous configurations were observed; however, there were some particles that were highly filamentous and were adhered to the mica in a configuration that crossed over itself; for example, a self-crossing particle would be a virion that bent into a full loop. This conformation is difficult to analyze via AFM imaging, because it becomes difficult to ascertain where the envelope of the virion starts and ends in the region that crosses over itself. In light of this, particles that did not cross over themselves were given preference over more constrained samples. This was because if the virion folds in on itself too closely, it is difficult to determine the exact location of the edge of the virion. PeakForce Tapping^®^ imaging of EBOVΔVP30 sample particles revealed 59 images of sample particles that were not self-crossing, and could therefore be analyzed topographically. Of these, the average length of the virion was 531.4 ± 264.6 nm, the average width of the virion was 42.6 ± 22.6 nm, and the average maximum height of the virion was 3.85 ± 3.53 nm. Unless specified otherwise, the reported measurement deviations are standard deviation.

To mitigate the use of infectious EBOV, many labs use rVSV-EBOV-GP as an EBOV proxy, in either an active or a UV-inactivated state. The use of rVSV as the base virion allows an increased degree of safety when handling, and the presence of EBOV GP on the surface allows the virion to roughly mimic the behavior of EBOV virus. The rVSV-EBOV-GP preparations are an important cornerstone of this characterization study due to rVSV-EBOV-GP’s frequent use in drug and vaccine development [7,8,9]. If the biomechanical surface characteristics of this approximating virion can be better assessed, it can inform observations in the past and future about the behavior of these particles in a drug development capacity.

When characterizing the rVSV-EBOV-GP, the AFM PeakForce Tapping^®^ mode revealed that the particles were semi-filamentous and exhibited a domed conformation when adhered to the treated mica substrate. A sample image of rVSV-EBOV-GP adhered to a mica substrate can be seen in Figure 1.

For the topographical analysis of rVSV-EBOV-GP, 240 images of particles adhered to mica were assessed. Of these, the average length of the virion was 285.3 ± 74.6 nm, the average width of the virion was 70.4 ± 20.3 nm, and the average maximum height of the virion was 7.4 ± 4.2 nm. This is a similar width and height to EBOVΔVP30, but the length is truncated, as expected from the naturally bullet-shaped rVSV virion. As the WT rVSV virion has average dimensions of 200 nm in length and 50 nm in diameter [13], the observed topographical measurements are within the expected size range of WT rVSV specimens.

Although rVSV-EBOV-GP is commonly used in EBOV therapeutic development, this virus is not typically commercially available. A common commercially available alternative is Sf9 EBOV VLP. PeakForce Tapping^®^ imaging yielded 54 images of Sf9 EBOV VLP particles on a treated mica surface. These revealed that the average length of the virion was 480.2 ± 152 nm, the average width was 115.1 ± 55.5 nm, and the average height was 51.5 ± 18.9 nm. This puts it in a similar dimensional category as the rVSV-EBOV-GP sample, with a height and width reminiscent of EBOVΔVP30, but a truncated length.

**Figure 2:**
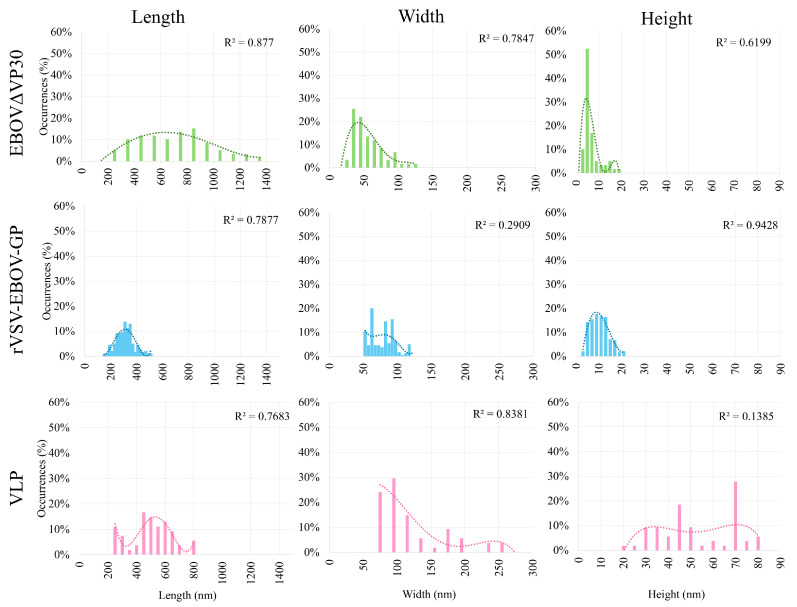
Histogram representation of characterization measurements of the sample particles. Each of the measurements is represented by a column, and each of the samples is represented by a row. Each histogram was fitted with a fourth-degree polynomial. This type of fitting was selected because, due to the samples being affixed to the mica substrate, it is possible that the virion may be attached to the mica in a relatively flat conformation, or it may be more upright. The application of a fourth-degree polynomial, rather than a Gaussian fit, allows for better fitting of the multiple peaks that appear.

**Figure A7:**
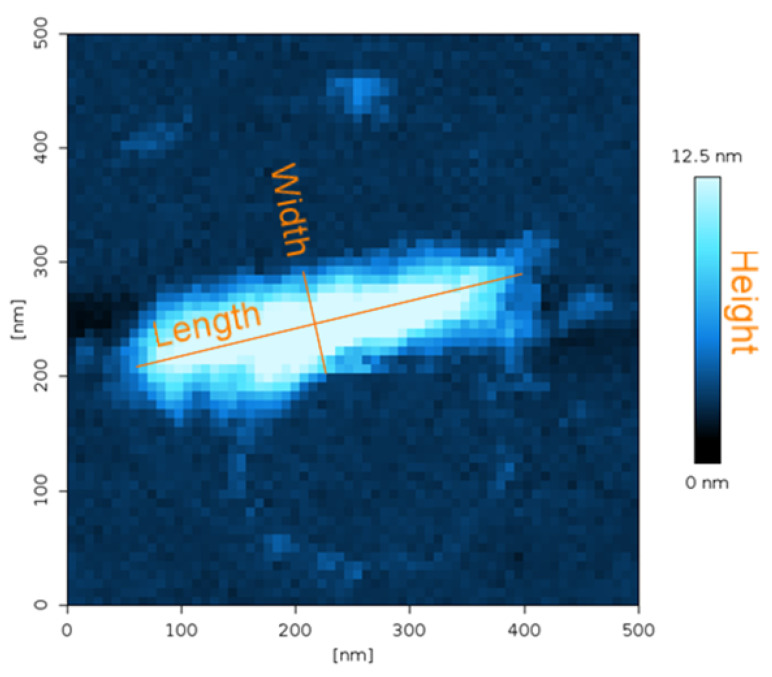
A sample image of rVSV-EBOV-GP with the dimensions of length, width, and height indicated. All other particles were processed in this same way.

The distributions of length, width, and height of each EBOV model are shown in Figure 2. For a depiction of the orientation of length, width, and height, see Figure A7.

### 2.2. Elastic Modulus of the Samples

Elastic modulus nanoindentation experiments were used to determine the elasticity of each sample virion. From these experiments, force curves with an indentation of less than 10% were used in order to minimize surface hardness interference from the mica substrate. From these, it was revealed that the elastic modulus of EBOVΔVP30 is 100.4 ± 51.5 MPa. Similarly, the elastic modulus of rVSV-EBOV-GP was determined to be 93.5 ± 22.3 MPa. While these two samples have an elasticity within an order of magnitude of each other, the VLP sample was observed to be quite stiff, with an elastic modulus of 420.0 ± 87.8 MPa. With each sample, indentations of less than 10% of the virion’s maximum height were used to prevent interference from the mica substrate. All other force scans were discarded.

**Figure 3:**
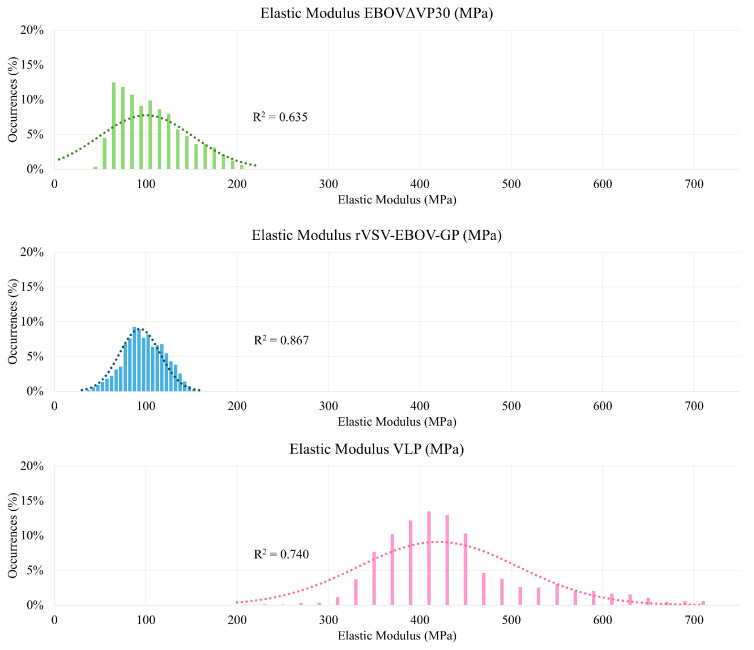
Histogram representation of elastic modulus measurements of the sample particles EBOVΔVP30, rVSV-EBOV-GP, and VLP.

The distribution of these nanoindentation curves can be seen in Figure 3. As shown, the elastic moduli of EBOVΔVP30 and rVSV-EBOV-GP are statistically similar, while the elastic modulus of the VLP is significantly higher. This is indicative of a much stiffer virion, which aligns with the rigid, straight virion shape observed in the topographical analysis. This may affect virion binding performance, as the natural EBOV is extremely flexible and therefore assumed to have a low elastic modulus in line with that of EBOVΔVP30 and rVSV-EBOV-GP.

**Figure 4:**
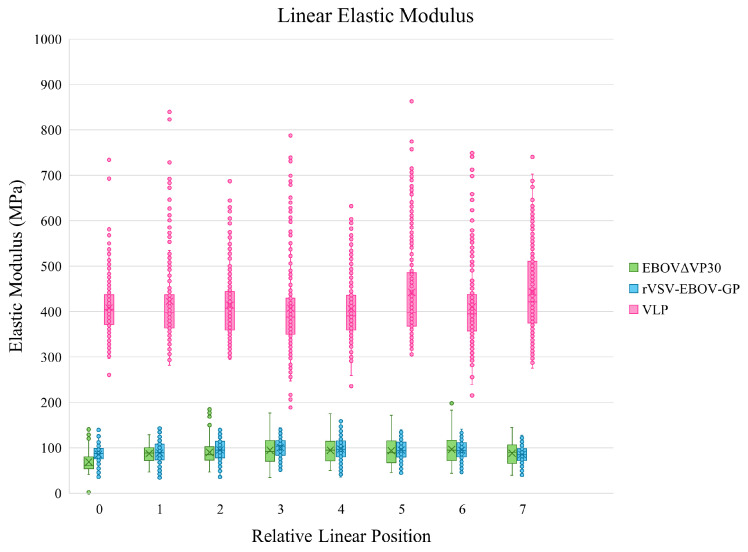
Box and whisker plot demonstrating the elastic modulus of three EBOVΔVP30 particles along their lengths. The Y axis represents the elastic modulus measurement at a given point, and the X axis represents the relative position of the cantilever tip along the length of each virion.

In addition to assessing overall virion elasticity, it is also important to determine whether the elasticity of each virion is uniform along its length, since each virion has a semi-filamentous structure. If the virion is uniformly stiff, then it behaves one way, but if there is variable stiffness along the length of the virion then virion movement and cell interaction could be highly impacted. To measure this, a series of force curves were taken at points along three distinct instances of each sample virion, and these were used to assemble a box plot representing elastic modulus along the length of each virion. This box plot can be seen in Figure 4.

As shown in Figure 4, the elastic modulus of each sample virion is relatively uniform along the length of the virion. Based on this, it is concluded that the elastic modulus of each of the sample particles is not dependent on the tip’s relative position along the center length of the virion.

Table 1: A summary of the results obtained from the EBOV-approximating virion characterization experiments. Length, width, height, and elastic modulus were measured with a nominal tip radius of 7 nm. Data presented are the geometric mean of each measurement ± one standard deviation.

Table 2: A summary of the number of scans used for each type of sample analysis. PeakForce Tapping^®^ scans were used for topographical analysis of the sample, and nanoindentation force curves were used for the elastic modulus analysis. Length, width, height, and elastic modulus were measured with a nominal tip radius of 7 nm.

## 3. Discussion

### 3.1. Physical Conformation of the Samples

With regard to physical conformation, the important characteristics to consider are the shape and size of the virion, as well as any twisting or bending that may be observed. EBOV typically has a filamentous structure with an approximate average length of 1000 nm, as confirmed by transmission electron microscopy [11]; however, the EBOV virion is highly pleomorphic, with many shape variants. When looking at the approximating particles, EBOVΔVP30 possessed a range of shapes, with many filamentous samples being bent or contorted in some way. There were also some spherical EBOVΔVP30 virions observed, resulting in a sample population that is heterogeneous in shape. Conversely, Sf9 EBOV VLP and rVSV-EBOV-GP possessed a semi-filamentous structure, where the particles were mostly straight, some with a slight bend in them. However, the structure of infectious EBOV can be capable of twisting into many configurations, such as six-shape or U-shape curves, as mentioned in the introduction to this study. This shaping of the filamentous EBOV is one of the critical features that defines EBOV. Due to the shorter conformation of the Sf9 EBOV VLP and rVSV-EBOV-GP particles, extensively curved or bent conformations were not observed. Therefore, it should not be assumed that these two particles can easily mimic the conformational distinctions between all the different subtypes of the EBOV disease. However, they are both a good representation of a generalized semi-filamentous shape that is similar in width and height to the EBOVΔVP30 sample. Additionally, the rVSV-EBOV-GP and Sf9 EBOV VLP samples may be able to represent the more spherical shapes of the EBOVΔVP30 sample reasonably well, depending on their elastic modulus and surface composition.

### 3.2. Elastic Modulus of the Samples

As posited by Witz et al. [15], the topography of a viral matrix is not enough to fully inform matrix behavior on its own. Instead, the mechanical properties of the matrix must be taken into account to fully explain the virus’ behavior in situ. In particular, the elasticity of EBOV approximating particles is of great interest, as the elasticity of the virion directly relates to its ability to bend into the complex conformations observed in infectious EBOV. Therefore, the elasticity of the EBOV viral matrix is posited to be low, to account for the high degree of bending which is typically seen, and a suitable approximating virion must have a similarly low elasticity. The elasticity of a virion can drastically affect the virion’s behavior and binding capability. It is important for an approximating virion to have an elastic modulus as close as possible to EBOV, as the elastic modulus could be important for viral uptake and entry. This shared elasticity allows the virion to emulate the virus as accurately as possible, which facilitates the development of drugs and therapies that are capable of successfully targeting infectious EBOV.

The elastic modulus of filamentous viruses in general is not widely characterized, but as the membrane characteristics are similar to those of spherical viruses, it is reasonable to assume that the elastic modulus of EBOV is in the same order of magnitude as spherical viruses. This assumption is made out of necessity, as most studies on virus virion elasticity that could be found focused on spherical virions [16], with the exception of a study on the elastic modulus of tobacco mosaic virus nanotubes [17]. In particular, there is a distinct lack of AFM nanoindentation studies on filovirus particles: to the best of our knowledge, this is the first study that includes AFM nanoindentation elastic modulus measurements of filovirus particles. Over a sample set of spherical viruses, elastic moduli have been shown to cover a range from 140 MPa to 1800 MPa [16], as determined by fitting sample force curves to an elasticity model, such as the Hertz model [18,19]. In keeping with this range, the elastic modulus of TMV has been shown to be in the range of 1.0 ± 0.2 GPa [17]. Therefore, an expected value for the elastic modulus of EBOV and EBOV-approximating particles is likely to fall somewhere within the range of 140 to 1800 MPa. With this in mind, we can consider the average elastic modulus of each approximating virion.

The analysis of elastic modulus for each virion was limited to force curves where the maximum indentation was 10% of the virion’s height or less, to prevent interference from the stiffness of the mica surface. The EBOVΔVP30 sample yielded an elastic modulus of 100.4 ± 51.5 MPa, which is on the same order of magnitude as a softer spherical virus [16]. Additionally, the EBOVΔVP30 sample demonstrated consistent elasticity along the length of the virion, indicating that the virion elasticity is not dependent on the relative position of the force curve. Similarly, the rVSV-EBOV-GP demonstrated an elastic modulus of 93.5 ± 22.3 MPa, which is aligned well with the elastic modulus of EBOVΔVP30. Linear analysis was also conducted on the rVSV-EBOV-GP virion, and the sample indicated a relatively consistent elastic modulus along the length of the virion, again showing that the elastic modulus at a certain point on the rVSV-EBOV-GP virion is not dependent on the distance from the semi-spherical ends of the virion. Finally, the Sf9 EBOV VLP demonstrated an elastic modulus of 420.0 ±87.8 MPa. This is within expectations for the literature values of elastic modulus for a spherical virion, but is noticeably stiffer than EBOVΔVP30 and rVSV-EBOV-GP. This higher virion elasticity could explain the rigid, cylinder-like shape observed via scanning AFM. It is also of note that the Sf9 EBOV VLP virion, being semi-filamentous, also demonstrates relatively consistent elasticity along its length, which shows that the elastic modulus at a certain position on the virion cannot be attributed to closeness to the curved outer edge of the virion.

There could be advantages and disadvantages to the elasticity of each virion. For example, if a study is not primarily focused on the conformational characteristics of a specific strain of EBOV, then the commercially available Sf9 EBOV VLP is a good choice, as the elasticity of the molecule matters less than the molecular interactions on the surface. As the preparation of EBOVΔVP30 is closest to the native infectious EBOV virion, it is assumed that the elasticity of infectious EBOV in the body is most similar to EBOVΔVP30. With this assumption in hand, it can be further observed that the rVSV-EBOV-GP virion is of a similar order of magnitude, but the IBT Bioservices VLP is a single order of magnitude stiffer. This is worth considering when designing an experiment that may be influenced by elasticity of the virion. However, if the study is contingent on the highly adaptable conformational qualities of EBOV, then the rVSV-EBOV-GP virion might be better suited, as the softer, more elastic surface might approximate for some of the conformational flexibility found in variants of EBOV.

## 4. Materials and Methods

### 4.1. Virion Production and Hypothetical Conformation

**Figure 5:**
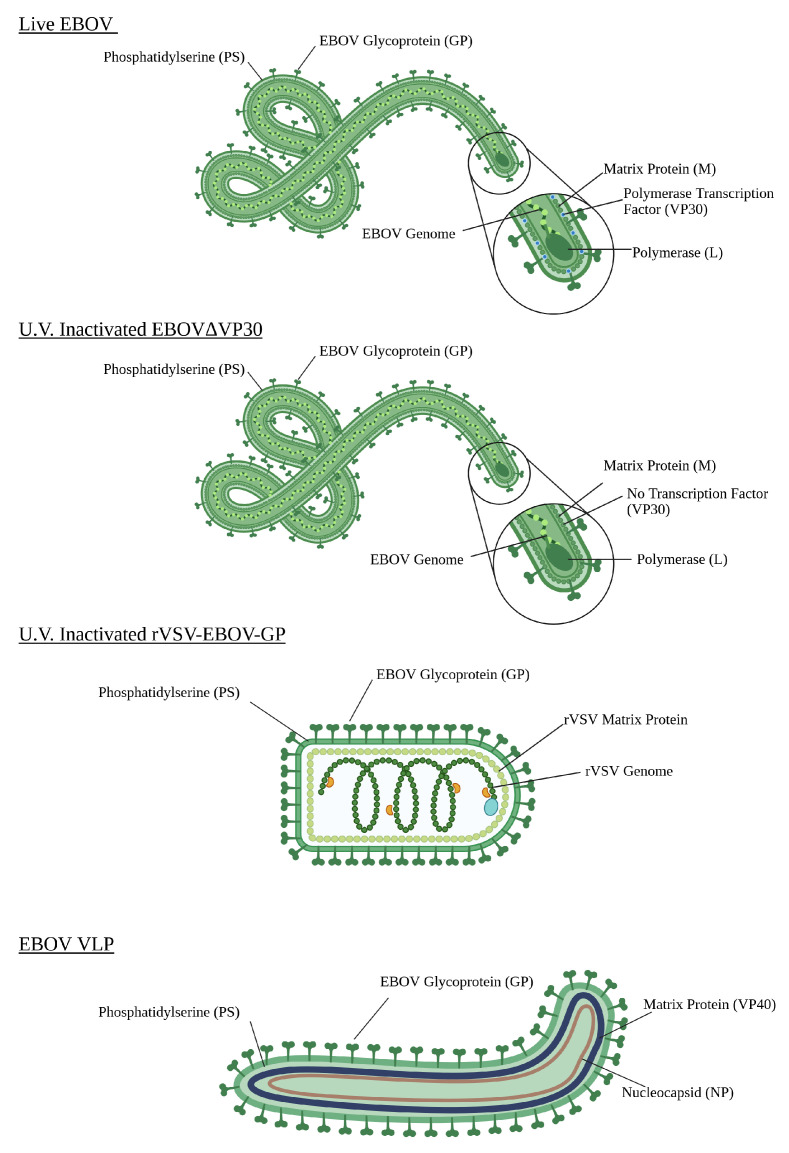
Schematic representation of the hypothetical physical conformation of infectious EBOV and the three approximating virions considered in this study. The EBOV virion shows the virus with a fully intact genome sequence, with abundant GP and PS on the surface of the viral envelope. The UV-inactivated EBOVΔVP30 sample shows a slightly altered version of infectious EBOV, where the VP30 segment of the viral genome was removed, causing there to be no production of VP30. The sample was also inactivated via UV light exposure. The UV-inactivated rVSV-EBOV-GP sample shows a standard rVSV virion, which does not encode the native G glycoprotein and was transfected to express EBOV GP on the virion surface instead. This allows it to mimic the surface properties of EBOV. This sample was also inactivated by UV exposure. The Sf9 EBOV VLP sample shows an engineered viral envelope, expressing GP and PS, and containing the EBOV proteins NP and VP40. This viral-like particle possesses no genome. Figure produced with Biorender.

The goal of this study is to acquire a highly precise characterization of the mechanical and structural properties of EBOV approximating particles. This is critical to the furthering of EBOV-targeting therapeutics because although the dimensions of EBOV virions are generally well characterized, much is still unknown about the mechanics of the EBOV virus. As is stated by Witz and Brown [15], the imaging of a viral matrix is static and is not enough to fully inform the behavior of the virion in situ. To better explain virus behavior in a host, a robust understanding of the mechanical properties of the viral matrix is critical. One such critical mechanical property of a matrix is its elasticity, as that informs the ability of the virus to bend and contort in the host. This could provide important implications as to the behavior of the virus—for example, a virus with a low elastic modulus (also known as Young’s modulus) would be more flexible, which would allow the matrix to bend and contort, as EBOV does. Therefore, the authors would posit that EBOV most likely has a low elastic modulus, to account for the high degree of conformational bending which is commonly observed. The authors would also posit, therefore, that a good approximating virion for EBOV must also have a similarly low elastic modulus. In order to determine the elasticity of each virion, the length, width, and height are first determined by Contact Mode Force Spectroscopy Nanoindentation to ensure that nanoindentation measurements can take place at the center of the virion. Then, elastic modulus nanoindentation measurements are conducted along the long axis of each virion at a shallow indentation depth to minimize substrate interference. This results in data that allow us to conclude the length, width, and height of each virion as determined by Contact Mode Force Spectroscopy Nanoindentation, and the elastic modulus of each virion as determined by nanoindentation. A comprehensive comparison between the different characteristics of each sample type is highly valuable for the advancement of therapeutic techniques designed to treat EBOV. For a schematic representation of each approximating virion, refer to Figure 5.

The first virion of interest to consider is the closest representation of infectious EBOV—EBOVΔVP30.

To derive EBOVΔVP30 from infectious EBOV, EBOV VP30-expressing Vero cells were infected with authentic EBOV at low MOI (0.005), from which supernatants were collected at 5 dpi. The supernatants were filtered through a 0.45-micron filter and purified by ultracentrifugation through a 20% sucrose cushion. The supernatant stocks of EBOVΔVP30 were then stored at −80 °C until needed. Virions used in this experiment were also inactivated by UV light exposure, which was achieved by placing the samples on ice for 30 min in a sterile container 15 cm from a UV light source in a biocontainment hood.

This is perfomed because VP30 is critical to EBOV’s ability to self-replicate [20], and therefore removing the VP30 segment inhibits the virus’s ability to self-replicate. This, in addition to other sterilization steps outlined by Halfmann et al. [21], makes the particles produced by this altered sequence safe for handling at a BSL-1 level. The viral plasmids are then sequenced to confirm the replacement of VP30 with GFP, and used to generate new sample particles. As the VP30 segment of the genome is replaced to produce these particles, this sample is referred to as EBOVΔVP30. Since the only modification to this virion is the replacement of VP30 with neomycin before budding, it can be assumed that the surface properties of EBOVΔVP30 are likely to be closely representative of infectious EBOV, with the potential for some small differences in physical conformation and PS distribution.

The second virion of interest to consider is recombinant replication-competent VSV expressing the EBOV GP and GFP in place of the G glycoprotein (rVSV-EBOV-GP). This virion is produced by inserting the EBOV GP gene upstream of an EGFP gene which replaced the G gene in the recombinant VSV genome. The virions are then produced in Vero cells using a low MOI (0.001) of the input virus and maintaining infection until a noticable cytopathic effect is acheived. The virions are then filtered through a 0.45-micron filter and stored at −80 °C until needed.

This virion, rVSV-EBOV-GP, is derived from the Indiana strain of VSV, and is a version of the rVSV-EBOV used as a clinical vaccine against EBOV [22,23,24]. This virion is widely used as a vaccine [22] and as a substitute for infectious EBOV in lab-scale experimentation [24]. This broad usage makes rVSV-EBOV a great candidate for this comparative study, as it is hypothesized that the mechanical properties (such as elasticity and PS distribution) are different between rVSV-EBOV and infectious EBOV. By investigating the nanomechanical characteristics of rVSV-EBOV, we can draw conclusions about their behaviors in experimental studies. Based on the construction of the rVSV-EBOV-GP [24], it is known that the surface of rVSV-EBOV-GP is rich in EBOV GP. Additionally, the presence of PS on the surface is confirmed in the virion via observed TIM-1 interaction [24]; however, the exact concentration of PS on the surface is unclear. Therefore, it is hypothesized that the rVSV-EBOV-GP virion will have the classic bullet-shaped conformation that is associated with rVSV, and the elasticity and PS-distribution on the surface of the virion are likely to be different, but within orders of magnitude of infectious EBOV and EBOVΔVP30.

In addition to these two commonly used surrogate viruses, there are limited commercially available options. One such option is the EBOV VLP that was obtained from IBT Bioservices (Rockville, MD, USA). This EBOV VLP is advertised as a specially engineered virion that emulates the surface properties and topography of the virus as closely as possible without introducing the dangerous level of infectivity that infectious viruses have. Briefly, the virion is produced via budding from Sf9 insect cells and is known to express NP, VP40 and GP, with no other internal components of EBOV being present. On the product specification sheet, the manufacturer provided a Western blot that proves the makeup they describe.

The type of cells that are used to bud the VLP from the original virus may have a dramatic effect on the shape, elasticity, and surface receptor density of the resultant VLP. The sample which was purchased from IBT Bioservices was produced via budding from Sf9 insect cells. Therefore, the VLP is referred to as Sf9 EBOV VLP, for increased clarity.

### 4.2. AFM Specifications

The JPK Nanowizard 4 AFM from Bruker (Camarillo, CA, USA) was employed to characterize each of the approximating particles. Briefly, AFM is a method of high-resolution microscopy that functions by contacting a nanometer size probe (the cantilever tip) to a surface (the sample) in order to ascertain the properties of the sample. If the tip is treated with a reactive substance, such as a binding agent, the interactions between the sample and the treated tip can also be observed. Unless otherwise specified, each virion type was observed while hydrated under 1x phosphate-buffered saline (1XPBS) to approximate the particles’ in vivo state. During analysis, three protein solutions were used: the EBOVΔVP30 protein solution, the rVSV-EBOV-GP protein solution, and the EBOV VLP protein solutions. All experiments were conducted at room temperature, approximately 298 K. Each of these particles is intended to imitate the membrane characteristics of EBOV, so they are well suited to comparative study. See Figure 5 for a schematic representation and description of infectious EBOV and each model viral virion.

### 4.3. AFM PeakForce Tapping^®^ Imaging

Each of the particles was examined via the Bruker PeakForce Tapping^®^ imaging mode, a proprietary mode owned by Bruker AFM that utilizes a variation of tapping mode. This mode averages a certain number of force scans per pixel to construct a topographical image of the surface of the sample. By this method, individual particles on the nanoscale can be imaged topographically—this is how the physical conformation of each virion was determined. Each sample was adhered to a fresh mica substrate treated with 10 μ g/mL poly-L-lysine and imaged under 100–200 μ L of 1× PBS to keep the sample from becoming dehydrated. The TESP-V2 AFM probe [25] was used for the bulk of topographical data collection, and the PEAKFORCE-HIRS-FA probe [26] was used to collect publication-quality image scans of each sample. Each cantilever was calibrated by the following steps: (1) aligning the laser on the center point of the tip, (2) adjusting the mirror to maximize the photodiode sum, (3) adjusting the laser detector to align with the center crosshairs on the JPK Nanowizard calibration screen, and (4) selecting the calibrate button in the calibration screen. The JPK Nanowizard 4 system has automatic calibration (as described), and fitting of the deflection equation to the curve was visually confirmed before each experiment.

### 4.4. Contact Mode Force Spectroscopy Nanoindentation

Another function of PeakForce AFM scanning is nanoindentation [27,28]. By this method, force scans can be conducted precisely over the location of a virion based on location coordinates determined by a PeakForce AFM image scan. This force scan registers a small indentation in the surface of the virion, and by analyzing the force and depth of the nanoindentation, the elastic modulus of the virion can be established. In this context, the elastic modulus is significant due to the influence of virion elasticity on virion behavior. If a virion is less elastic, it may require a specific conformation to bind appropriately to a cell receptor, but if a cell is highly elastic, it may be capable of conformational bending that would allow it to easily bind to one or more receptors, with less concern about positioning. To minimize interference from the mica substrate, nanoindentation depths less than 10% of the maximum height of the observed virion were used in the analysis. Any indentations deeper than this are assumed to be affected by the stiffness of the mica substrate and have been discarded from the dataset. For specific sample preparation and imaging protocols, see Appendix A. The TESP-V2 AFM probe was used for elastic modulus nanoindentation data collection, and each cantilever was calibrated by the automatic JPK Nanowizard calibration process, as described in Section 4.3. Additionally, the automatic fitting of the deflection equation to the curve was visually confirmed before each experiment.

### 4.5. Single-Molecule Force Spectroscopy

One of the key tenets of authentic EBOV is the presence of EBOV GP on the surface of the virion. This receptor allows the virus to penetrate the host cells through GP-mediated host cell attachment. Each of the EBOV model particles being assessed are documented to have EBOV GP on the surface. To confirm this, the anti-EBOV mAb KZ52 was used to conduct single-molecule force spectroscopy. By measuring the unbinding force between the KZ52 antibody and each model virion’s surface, it is possible to confirm the presence of EBOV GP on the virion surface. Results of this experiment can be found in Appendix A.4.

## 5. Conclusions

Characterization of EBOV-approximating particles is critical to the ongoing development of therapeutic drugs to combat the deadly EBOV disease. Overall, rVSV-EBOV-GP demonstrated a physical configuration and elasticity more aligned with the filamentous shape and elasticity of EBOVΔVP30 and the known stiffness of other spherical viruses, while VLP had a similar semi-filamentous shape but exhibited a stiffer virion elasticity. Additionally, all three approximating particles have EBOV GP present on the virion surface, equipping them to mimic native EBOV surface properties.

Based on these conclusions, EBOVΔVP30 is best suited to studies dedicated to the development of EBOV therapies and treatments due to its close approximation of infectious EBOV. If EBOVΔVP30 cannot be obtained, however, both the rVSV-EBOV-GP and IBT Bioservices EBOV VLP are well suited to conducting experiments investigating the nanomechanical interactions between the surface of EBOV and various molecules, thereby forwarding the development of more effective medications to treat this deadly disease.

Further work on this subject is to be focused on the assessment of each virion’s ability to enter in vitro host cells transfected with known and suspect EBOV entry receptors in order to examine whether each virion is capable of utilizing EBOV internalization pathways. Included in this is the assessment of each virion’s ability to adhere to EBOV GP and PS receptors, as this is a critical component of EBOV internalization.

## Figures and Tables

**Table 1:** Results Summary.

Sample	Length (nm)	Width (nm)	Height (nm)	Elastic Modulus (MPa)
infectious EBOV *	805	80	80	–
EBOVΔVP30	531.4 ± 264.6	42.6 ± 22.6	3.85 ± 3.53	100.4 ± 51.5
rVSV- EBOV-GP	285.3 ± 74.6	70.4 ± 20.3	7.4 ± 4.2	93.5 ± 22.3
Sf9 EBOV VLP	480.2 ± 152	115.1 ± 55.5	51.5 ± 18.9	420.0 ± 87.8

* infectious EBOV values according to Klenk and Feldmann, 2004 [14].

**Table 2:** Number of Scans Used in Analysis.

Sample	Number of Imaging Scans	Number of Nanoindentation Force Curves
EBOVΔVP30	59	7242
rVSV- EBOV-GP	204	1972
Sf9 EBOV VLP	54	2531

**Figure A1:**
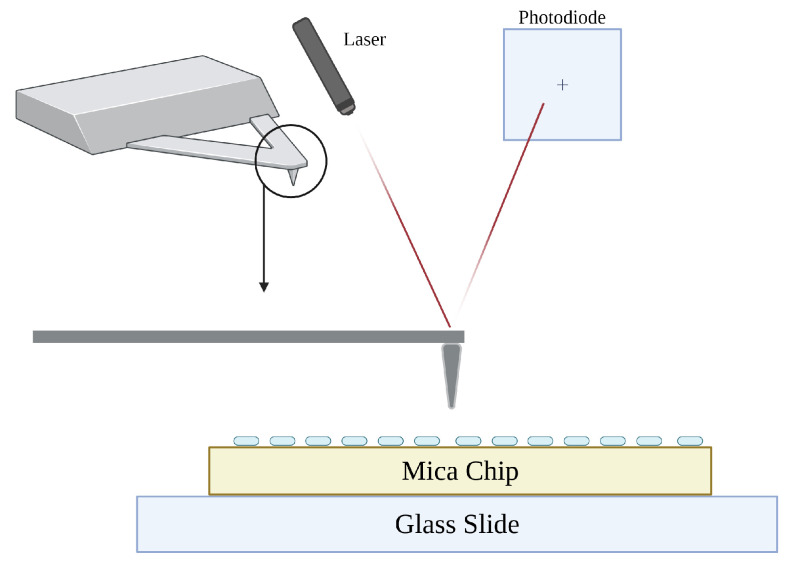
Representation of the PeakForce Tapping^®^ Imaging method. The cantilever scans over a surface of interest, and the laser deflections allow the software to convert tip deflection into a map of surface height. Figure produced in Biorender.

**Figure A2:**
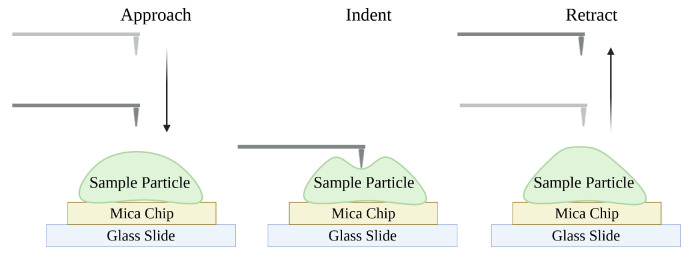
Schematic representation of a Contact Mode Force Spectroscopy Nanoindentation experiment of a generic sample. The tip indents the virion of interest with a certain amount of force, and the rate and extent of indentation can be used to determine the elastic modulus of the virion of interest. The cantilever approaches the surface, indents the sample, and retracts. As the tip is untreated, there is no snap-in or adhesion to be seen here. Figure produced in Biorender.

**Figure A3:**
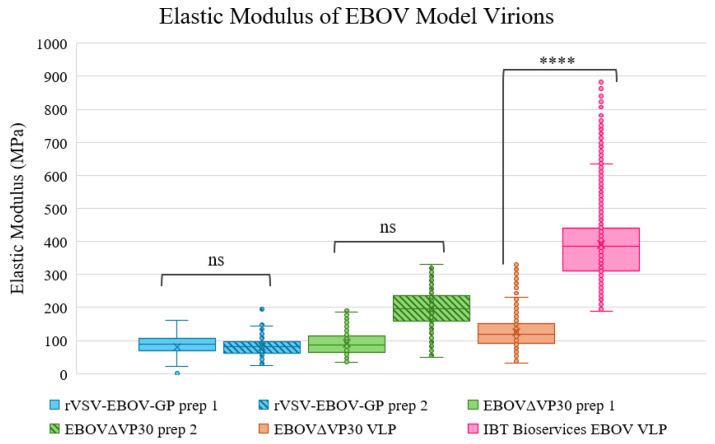
A box plot of the elastic modulus nanoindentation measurements for each viral preparation under consideration. There are two preparations each of rVSV-EBOV-GP and EBOVΔVP30, and there is a single preparation of Vero EBOV VLP and Sf9 EBOV VLP. The “****” notation indicates that there is *p* < 0.0001 significance between each of the two different populations of Sf9 EBOV VLP and Vero EBOV VLP, indicating a statistically significant difference between the two samples. The “ns” notation between the rVSV-EBOV-GP groups and the EBOVΔVP30 groups indicates a *p* > 0.05, so there is not a statistically significant difference between each of the two groups.

**Figure A4:**
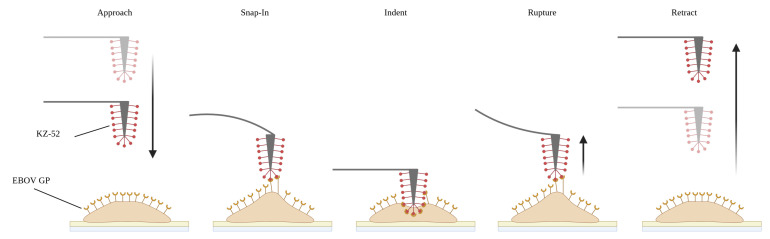
A schematic representation of the path the cantilever takes to the sample during force spectroscopy measurement. Image produced in BioRender.

**Figure A5:**
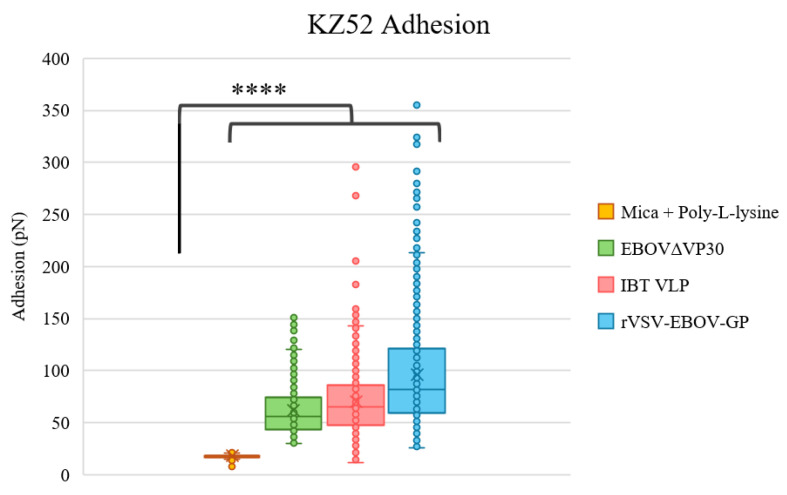
A box plot showing the KZ52 adhesion force on the substrate and each of the EBOV model virions. The “****” notation reflects a *p* < 0.0001 between the poly-L-lysine treated mica and each of the virions of interest. This indicates that there is specific KZ52 adhesion occurring on the virions.

**Figure A6:**
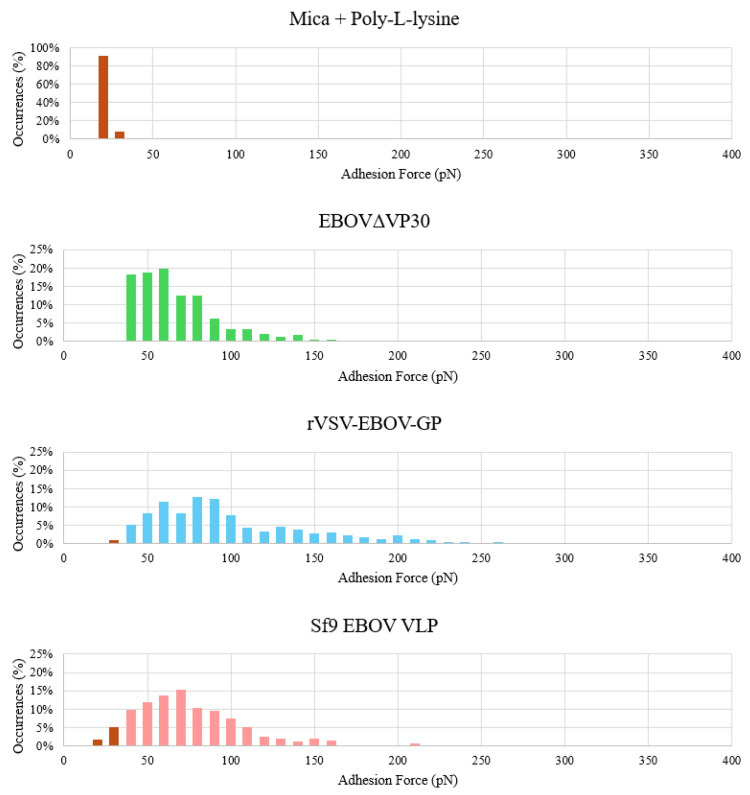
A series of histograms showing the distribution of adhesion forces between KZ52 and each of the sample virions. In instances where the bins of the samples overlap with the bins of the substrate, those sample bins are colored to match the Mica + Poly-L-lysine histogram.

**Figure A8:**
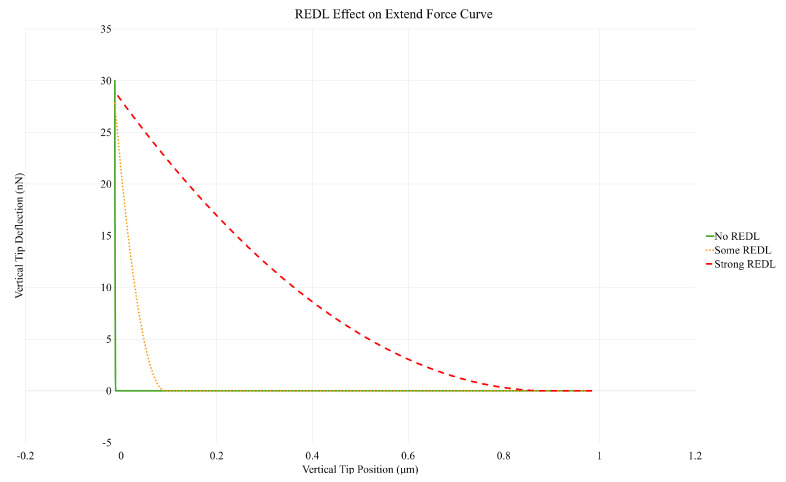
A schematic representation of three force curves representing the effect of REDL on an AFM force curve.

**Figure A9:**
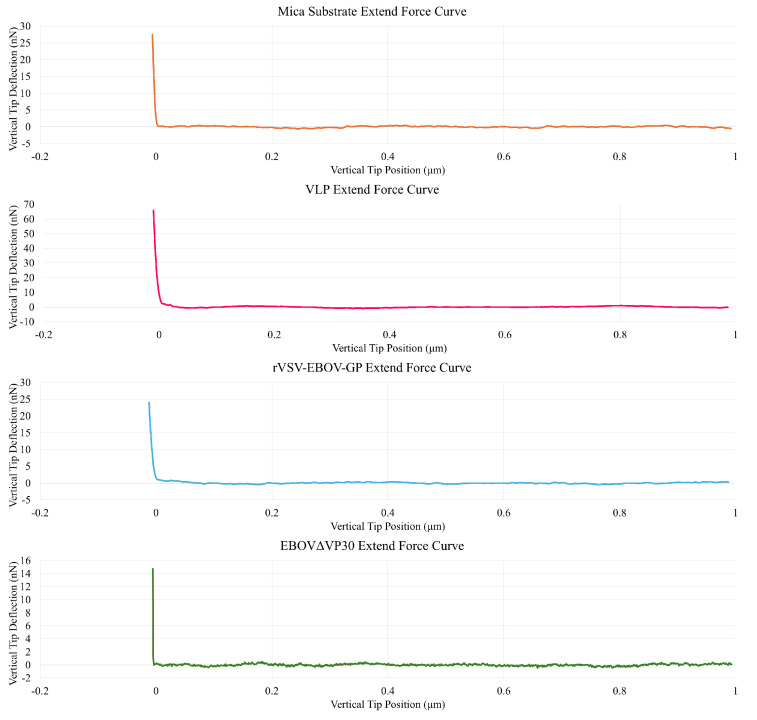
A representative force curve from each of the three samples. Each scatterplot depicts the extend curve from an AFM nanoindentation measurement of the sample.

## Data Availability

Data is contained within the article and Supplementary Material.

## Supplementary Materials

The following supporting information can be downloaded at: https://www.mdpi.com/article/10.3390/ijms26115185/s1, a list of the supplemetal videos which are available in tandem with this publication in order to support the description of each EBOV model virion. Video S1. A 3D representation of the Peakforce AFM image of an EBOVΔVP30 shown in Figure 1. Video S2. A 3D representation of the Peakforce AFM image of an rVSV-EBOV-GP shown in Figure 1. Video S3. A 3D representation of the Peakforce AFM image of a VLP shown in Figure 1.

## Abbreviations

The following abbreviations are used in this manuscript:

AFM	Atomic Force Microscopy
EBOV	Ebolavirus
VLP	Virus-Like Virion
GP	Glycoprotein
PS	Phosphatidylserine
rVSV-EBOV-GP	Recombinant Vesicular Stomatitis Virus Transfected with EBOV Glycoproteins
EBOVΔVP30	UV-Inactivated Ebolavirus with the VP30 Segment of the RNA Sequence Replaced with Neomycin.
EBOV-VLP	Ebola Virus-Like Particles Obtained from IBT Bioservices in 2022

## Appendix A. Supplemental Methods and Materials

This appendix contains further explanations of the methods and materials which were briefly alluded to in the main body of the text. They are expanded upon in further detail here in order to avoid detracting from the momentum of the paper.

### Appendix A.1. PeakForce Tapping® Imaging

PeakForce Tapping^®^ Imaging mode is a proprietary AFM imaging mode designed by Bruker to produce high-resolution AFM images. This mode was used to determine the morphological characteristics of each sample. This mode functions by utilizing the tapping mode to break down the scanning range into pixels and then averaging the values from each pixel to determine a more accurate characterization of the virion. Features on the molecular and atomic scale can be observed with this method.

### Appendix A.2. Contact Mode Force Spectroscopy Nanoindentation

Contact Mode Force Spectroscopy Nanoindentation is an AFM method where the AFM tip is indented into a virion with a certain amount of force. The resulting force curve can be analyzed by a Hertz model (Equation (A1)) [18] that assumes little adhesion between the virion of interest and an untreated AFM tip, and from this model the elastic modulus of the virion of interest can be determined.

(A1) $$\begin{array}{cc} E & =\frac{3FR}{4a^{3}} \end{array}$$

*F* = nanoindentation force (F), *R* = sphere radius of the nanoindenter (m), *a* = contact radius between the two surfaces.

It is important to take into consideration the depth of nanoindentation. To be statistically significant, indentation depth must be at least 1–2 nm. However, if the indentation depth is greater than 10–15% of the overall height of the virion of interest, then interference from the substrate material begins to overwhelm the nanoindentation measurement, resulting in a false increase in the stiffness of the virion of interest. To minimize interference of the substrate material, only nanoindentation curves that had an indentation depth between 1 and 10 nm were used in nanoindentation analysis.

### Appendix A.3. Uniformity of Elastic Modulus Across Viral Preparations

To verify whether the measured elastic modulus of each model virion was characteristic of the model or whether it was highly dependent on the viral preparation batch from which the sample came, we tested a second preparation of the rVSV-EBOV-GP and EBOVΔVP30 samples, each of which was prepared in the same manner as the initial samples of each virion. We were also able to procure a second type of EBOV VLP which was produced via budding from Vero cells to determine whether the type of cell used in budding would dramatically affect the elastic modulus of the EBOV VLP. This new VLP is referred to as Vero EBOV VLP for clarity.

Each of the three secondary samples underwent elastic modulus nanoindentation, and the results revealed that while there was no statistically significant difference between the different preparations of rVSV-EBOV-GP and EBOVΔVP30, there was a dramatic difference between the elastic moduli of the two different EBOV VLPs. This was not unexpected: while the rVSV-EBOV-GP and EBOVΔVP30 samples were produced in the same manner, the EBOV VLPs were not. However, it is notable that the elastic modulus of the Vero EBOV VLP is much closer to that of rVSV-EBOV-GP and EBOVΔVP30 than the Sf9 EBOV VLP. This may be because other than the Sf9 EBOV VLP, all samples were produced via budding from mammalian cells. It is possible that the use of insect cells in EBOV VLP production is responsible for yielding a stiffer EBOV VLP; however, it is difficult to determine definitively. Regardless, the elastic modulus of each virion seems to be a characteristic of the method of viral preparation and appears to be consistent across multiple batches. For a box plot representation of the results, see Figure A3.

### Appendix A.4. Single-Molecule Force Spectroscopy

All of the model virions examined shared the common feature of surface expression of the EBOV GP. We probed the interaction between this antigen and the well-characterized monoclonal antibody KZ52 [29] with single-molecule force spectroscopy. In this technique, an AFM cantilever functionalized with KZ52 repeatedly approaches and withdraws from the virion, enabling individual antibody–antigen bonds to form and rupture. The peak rupture force recorded in each cycle provides a quantitative measure of bond strength. Similar assays have been employed by other groups to verify GP presentation on EBOV model virions [30,31,32].

Force scans on EBOV model virions were compared to force scans on the poly-L-lysine-treated mica substrate to demonstrate that a statistically significant adhesion is present between the KZ52 tip and each sample. A schematic representation of the force pulling steps can be seen in Figure A4.

As can be seen in Figure A4, the tip first approaches the virion, and upon moving close to the virion surface, electrostatic interactions between the virion and KZ52 cause the cantilever to bend towards the sample. This behavior is referred to as the ”snap-in”. Then, minor indentation occurs, and the cantilever begins to retract. As the cantilever retracts, the newly formed bonds between the KZ52 and the virion are pulled taught, bending the cantilever down. Once the force exerted on the bond exceeds the adhesion force, the bond ruptures, and the cantilever continues to ascend to the top of the current z-motor position. This process is repeated several thousand times over 10–20 virions from each sample, which yields the body of adhesion data presented in this study. Note that force pulling is conducted in 1XPBS with the addition of 1 wt% fat-free BSA to minimize nonspecific tip-surface interactions. Force pulling was conducted on each of the sample particles, as well as the poly-L-lysine treated mica substrate. The mica substrate, which does not express EBOV GP and therefore serves as a negative control, displayed significantly lower adhesion, reflected by both the mean adhesion force and the frequency of adhesion events recorded with the KZ52-functionalized tip compared to the virions (Table A1; Figure A5 and Figure A6).

Table A1: A summary of the results obtained from the single-molecule force spectroscopy unbinding between KZ52 and each of the model virions, with the mica + poly-L-lysine substrate included as a reference. Adhesion is the mean value ± a single standard deviation, and adhesion frequency is a percentage representing the fraction of force curves with single-molecule adhesion over the total number of force curves per sample. Adhesion force curves were taken with a KZ52 functionalized MLCT-BIO-DC-C tip.

**Table A1:** KZ52 Adhesion.

Sample	Adhesion Force (pN)	Adhesion Frequency (%)
Mica + Poly-L-lysine	17.9 ± 1.9	6.1%
EBOVΔVP30	58.0 ± 24.5	12.1%
rVSV- EBOV-GP	85.2 ± 51.2	16.2%
Sf9 EBOV VLP	62.9 ± 33.9	18.6%

### Appendix A.5. Sample Preparation

For the experiments in this study, both VLPs were prepared for AFM scanning in the same way. Freshly cleaved mica sheets were treated with 10 μg/mL poly-L-lysine in DIH2O for 30 min. Following this, the mica sheet was rinsed in DIH2O and dried with compressed air ten times. At this stage, a protein solution of virion and 1XPBS (1:400–1:800) was deposited onto the surface of the mica. VLP particles were allowed to diffuse to the mica surface for 30 min, and then the sample was gently rinsed with 10 mL of 1XPBS and immediately moved to the AFM to begin scanning. For imaging and elastic modulus measurement, 1XPBS was used, and for adhesion measurements 1XPBS was supplemented with 1 wt% fat-free BSA.

### Appendix A.6. Explanation of Topographical Measurements

In this study, the terms “length”, “width” and “height” are used frequently to describe the sample virion in three-dimensional space. These measurements are relative to the surface of the mica substrate, with length being the largest lateral measurement, width being the smaller lateral measurement, and height being the maximum distance between the outer edge of the virion and the mica surface. Figure A7 is attached for further elucidation of these measurements.

### Appendix A.7. Acknowledgement of the Repulsive Electrical Double Layer Effect

Within the field of AFM, there is a phenomenon known as the repulsive electrical double layer (REDL) effect. This is an effect whereby a charged surface, such as the negatively charged virus, may exert a small electrical force on its surroundings in an environment which can be up to nN magnitude [33]. This effect can be of great concern when low imaging forces are required, such as probing DNA.

As shown in the schematic curves on Figure A8, the higher the force of the electronic field, the greater the area under the extension force curve. This can potentially interfere with height scanning measurements, if the scan is significantly disturbed, as in the case of Strong REDL shown in red on Figure A8. To determine whether the REDL effect has a significant impact on the data in this study, a set of force curves from each sample was visually analyzed to determine the significance of REDL on the measurement of each sample virion. A representative force curve from each sample can be seen in Figure A9. As can be seen, the force curve from each sample demonstrates very little presence of REDL, in comparison to the overall force being exerted by the probe. Therefore, the authors posit that while some REDL effect may be present, it is not of significance to the measurements of topography and elasticity of each sample.
